# Supplementary material for: Saccharomyces boulardii (CNCM I-745) improves intestinal damage in sepsis by remodeling intestinal flora structure
Source: Front Cell Infect Microbiol. 2025 Jul 31;15:1602792. doi: 10.3389/fcimb.2025.1602792 (PMC12350329; doi:10.3389/fcimb.2025.1602792)
Supplement: Supplementary file 1 [file DataSheet1.zip › summary/summary/8_Advanced_analyse/2_PICRUSt2_result/COG/Group.DW0_vs_BW0/PICRUSt2_COG_Group.DW0_vs_BW0_diff.pdf]

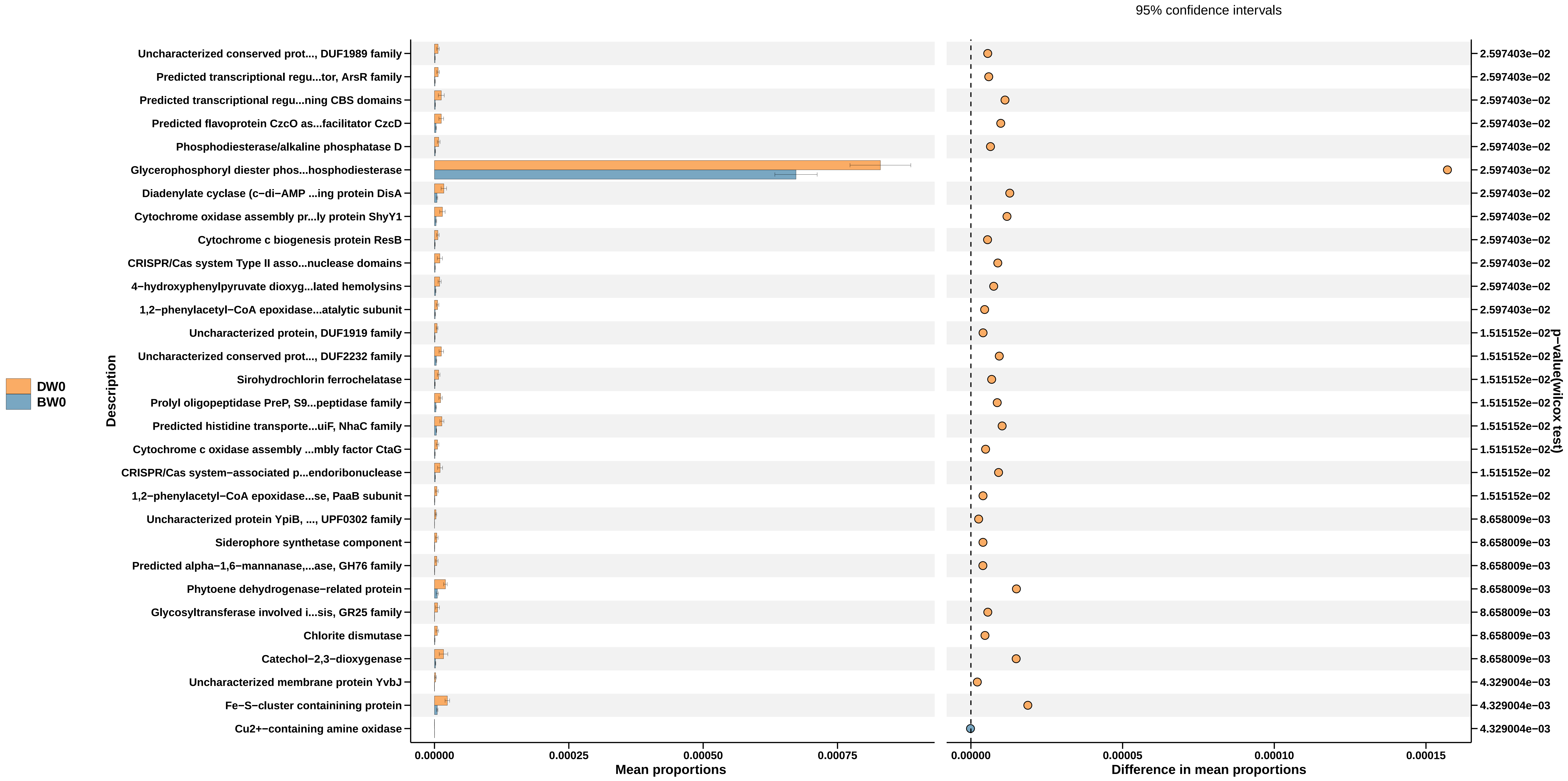
95% confidence intervals
DW0
BW0
Description
Uncharacterized conserved prot..., DUF1989 family
Predicted transcriptional regu...tor, ArsR family
Predicted transcriptional regu...ning CBS domains
Predicted flavoprotein CzcO as...facilitator CzcD
Phosphodiesterase/alkaline phosphatase D
Glycerophosphoryl diester phos...hosphodiesterase
Diadenylate cyclase (c−di−AMP ...ing protein DisA
Cytochrome oxidase assembly pr...ly protein ShyY1
Cytochrome c biogenesis protein ResB
CRISPR/Cas system Type II asso...nuclease domains
4−hydroxyphenylpyruvate dioxyg...lated hemolysins
1,2−phenylacetyl−CoA epoxidase...atalytic subunit
Uncharacterized protein, DUF1919 family
Uncharacterized conserved prot..., DUF2232 family
Sirohydrochlorin ferrochelatase
Prolyl oligopeptidase PreP, S9...peptidase family
Predicted histidine transporte...uiF, NhaC family
Cytochrome c oxidase assembly ...mbly factor CtaG
CRISPR/Cas system−associated p...endoribonuclease
1,2−phenylacetyl−CoA epoxidase...se, PaaB subunit
Uncharacterized protein YpiB, ..., UPF0302 family
Siderophore synthetase component
Predicted alpha−1,6−mannanase,...ase, GH76 family
Phytoene dehydrogenase−related protein
Glycosyltransferase involved i...sis, GR25 family
Chlorite dismutase
Catechol−2,3−dioxygenase
Uncharacterized membrane protein YvbJ
Fe−S−cluster containining protein
Cu2+−containing amine oxidase
0.00000
0.00025
0.00050
0.00075
Mean proportions
0.00000
0.00005
0.00010
0.00015
Difference in mean proportions
p−value(wilcox test)
2.597403e−02
1.515152e−02
8.658009e−03
4.329004e−03
